# Neuromedin B and Its Receptor: Gene Cloning, Tissue Distribution and Expression Levels of the Reproductive Axis in Pigs

**DOI:** 10.1371/journal.pone.0151871

**Published:** 2016-03-24

**Authors:** Zhiyu Ma, Juan Su, Tingting Guo, Mengmeng Jin, Xiang Li, Zhihai Lei, Yuanlong Hou, Xiaoliang Li, Cuicui Jia, Zheng Zhang, Ejlal Ahmed

**Affiliations:** College of Veterinary Medicine, Nanjing Agricultural University, Nanjing 210095, PR China; University of Rouen, FRANCE

## Abstract

Neuromedin B is one member of a family of bombesin-like peptides, which performs a variety of physiological functions via their receptor (NMBR) in most mammals. However, the genes encoding NMB and NMBR and their functions especially reproduction of the pigs are currently not fully understood. To research the physiological functions of NMB, we cloned and analyzed the NMB and NMBR genes, and systematically investigated the expression levels of NMB and NMBR mRNA using relative real-time PCR and the distribution of NMBR by immunohistochemistry (IHC). Experimental results show that the sequences of the amino acid and gene of NMB and NMBR were highly conservative and homology in many species, Significantly, the relative RT-PCR results revealed that NMB was mainly expressed in the central nervous system (CNS), whereas NMBR is highly expressed in peripheral tissues and organs, such as endocrine tissues, glands and reproductive organs. The IHC results show that NMBR positive cells were widely distributed in the body, such as respiratory and circulatory system, digestive system, urogenital system, in lymphatic organs and in the endocrine system. We also systematically investigated expression levels of NMB and NMBR in the reproductive axis using relative real-time PCR. In sow estrous cycle, the hypothalamic levels of both NMB and NMBR mRAN were similar, but the expression levels of the pituitary were negatively correlated. Expression levels in the ovarian system are lowest in metestrus phases and highest in proestrus and estrus phases. In boar post-natal development stages, the hypothalamic, pituitary and testicular levels of NMB and NMBR mRNAs showed developmental changes on postnatal day 30, 60, 90 and 120. Taken together, this study provided molecular and morphological data necessary for further research of physiological function of NMB/NMBR system in the pigs.

## Introduction

Neuromedin B (NMB) is one member of a family of bombesin-like peptides in mammals, which are decapeptides that were originally identified in porcine spinal cords [1]. In 1988, Krane et al. isolated human cDNA encoding NMB by screening human hypothalamic libraries [2] and identified the molecular structure of human NMB. Subsequently, the NMB gene was isolated from rats, and a 117-amino acid prepro-NMB was revealed via nucleotide sequence analysis [3]. Since its discovery, NMB has been found to be widely expressed in central nervous system (CNS) and in peripheral organs via RT-PCR and in situ hybridization. In humans, NMB is mainly expressed in the hypothalamus, stomach and colon and to a lesser degree in the cerebellum, pancreas and adrenal glands [2]; in adipose tissues [4]; and in the urinary tract [5]. In rats, NMB mRNA has been prominently identified in the dentate gyrus, olfactory bulb, dorsal root ganglion and brain stem [3,6]. In peripheral tissues and organs, NMB has been found in the esophagus, stomach, intestines, uterus, urinary bladder, lungs, gall bladder, adipose tissues, gastrointestinal tissues, pancreas and pituitary [4,7,8].

It has been shown that the Neuromedin B receptor (NMBR) is part of the G protein-coupled receptor (GPCR) family, which when activated by NMB performs a variety of physiological functions. The amino acid sequence of the NMBR is well conserved across various species [6]. Expression levels of NMB receptor mRNA have been reported for humans, rats, mice, and monkeys [9–12]. High expression levels of NMBR mRNA are found in the CNS and in several peripheral tissues. In the CNS, the NMBR is widely expressed in various brain regions, including the caudate nucleus, amygdala, thalamus, hippocampus, brain stem, hypothalamus, spinal cord and olfactory region in rats and mouse. In peripheral tissues, broad distribution of the NMBR has been found in the testis, urogenital smooth muscles, gastrointestinal system, esophagus and adipose tissues [4,8,10–13]. Moreover, NMB receptors have been found on various types of tumors, including CNS tumors, small cell and nonsmall cell lung cancers, carcinoids (intestinal, thymic, bronchial), human pancreatic cancer cell lines and ovarian epithelial cancers [14,15]. NMB/NMBR is an important physiological regulator of smooth muscle contraction [6,13,16]. It also plays a role in behavior (especially fear and anxiety [17–19], stress, itching and scratching behavior [20–23]), feeding [20,24,25], thermoregulation [26], thyrotropin release inhibitors [27,28], reproduction [29], blood pressure, sucrose regulation, energy balance and cell growth [4,6]. NMB exerts its functions through activation of intracellular signaling pathways. For example, NMB and its receptor can induce angiogenesis via ERK and Akt activation in endothelial cells [30] and also induce labor via a RELA/IL6-mediated pathway [31].

After mice and rats, pigs are the third most commonly used experimental animal. Pigs represent a special species that may serve as an appropriate model for human biomedical research and as an application-appropriate model for studies on reproduction [32]. As non-primate mammals, pigs have a body size, anatomy and physiology most similar to that of humans [33]. In addition, pigs play an important role in animal husbandry. Therefore, we use pigs as a model to study molecular and cellular mechanisms through which the NMB/NMBR system performs physiological functions during reproduction. A previous study has shown that NMB can stimulate the HPG axis via hypothalamic GnRH in male rats [29]. Whether pig NMB performs the same function during reproduction requires further examination.

To determine physiological functions of NMB and its potential role, we first cloned and analyzed pig NMB and NMBR genes, systematically investigated relative expression levels of NMB and NMBR mRNA via relative real-time PCR. We analyzed the distribution of NMBR in various tissues using immunohistochemistry (IHC). We also systematically studied expression patterns of NMB and NMBR mRNA on the reproductive axis of the sow estrous cycle and boar post-natal development stages. Collectively, these results describe anatomical locations of NMB and NMBR in pigs and present molecular and morphological data necessary to further investigate physiological functions (especially reproductive functions) of NMB/NMBR, which was the purpose of our ongoing study.

## Materials and Methods

### Ethics statement

All animals were fed based on Chinese Local Pigs breeding standards. All experimental protocols were reviewed and approved of by the regional Animal Ethics Committee of Nanjing Agricultural University under project number 2009ZX08009-143B. Sampling procedures used strictly complied with the Guidelines on Ethical Treatment of Experimental Animals (2006) No. 398 set by the Ministry of Science and Technology, China. Throughout the experimental session, pigs were housed under constant conditions and were carefully nursed to limit their exposure to stress. Each process was conducted in strict accordance with animal protection committee regulations to minimize risks of injury.

### Animals and treatment

Animals used for the RT-PCR studies: six Xiaomeishan pigs (male: n = 3; female: n = 3) that were 30 d old and that weighed 10 ± 2 kg were used. The pigs were terminally anesthetized via an intraperitoneal (ip.) injection of 20% urethane (5 mL/kg body weight), were decapitated within 15 min after anesthesia took effect (i.e. animals lost conciseness and whole body muscle relaxation was observed), and 40 tissues were collected: the cerebral cortex, cerebellum, spinal cord, medulla oblongata, pons, midbrain, hypothalamus, hippocampus, olfactory bulb, hypophysis, heart, liver, spleen, lung, kidney, stomach, duodenum, jejunum, ileum, cecum, colon, rectum, parotid gland, mandibular gland, thymus, adrenal gland, pancreas, bone marrow, tonsil, jejunum lymph nodes, testis, epididymis, ovary, uterus, esophagus, trachea, aorta, bladder, fat and muscle. The tissues were removed and stored at −70°C until being used for mRNA extraction. All pigs were free from overt signs of disease during tissue collection.

Six Xiaomeishan pigs were examined for the immunochemistry study as described above. The pigs were anaesthetized with an intraperitoneal injection of 20% urethane (5 mL/kg body weight), and then the tissue fixation and preparation procedures were performed as reported above [34]. Peripheral tissues were sampled and postfixed with the 4% paraformaldehyde fixative and were stored until further paraffine embedding was performed [35].

Animals examined for the estrous cycle study: 16 large, white and virginal cross-bred pigs of 90 ± 5 kg in weight were examined. The estrous cycles were monitored via vaginal smears, and only pigs that had at least three consecutive 21-d cycles were used [36,37]. All pigs were classified into four groups: proestrus (day 18 of the estrous cycle, n = 4), estrus (day 20 of the estrous cycle, n = 4), metestrus (day 2 of the estrous cycle, n = 4) and diestrus (day 14 of the estrous cycle, n = 4). The pigs were terminally anesthetized using an intraperitoneal (ip.) injection of 20% urethane (5 mL/kg body weight) and were decapitated within 15 min after anesthesia took effect (i.e. animals lost conciseness and whole body muscle relaxation was observed) on the scheduled days as shown above. Then, the hypothalamus, pituitary, and ovaries were removed and frozen at −70°C before being used for RNA extraction.

Animals examined for the post-natal development study: fifteen male Xiaomeishan pigs were utilized. The pigs were classified into five groups based on the dates on which they were euthanized: postnatal day 3, day 30, day 60, day 90, and day 120 (n = 3 per group). The pigs were terminally anesthetized using an intraperitoneal (ip.) injection of 20% urethane (5 mL/kg body weight) and were decapitated within 15 min after anesthesia took effect (i.e. animals lost conciseness and whole body muscle relaxation was observed) on the scheduled days as described above. Then, the hypothalamus, pituitary and testis were removed and frozen at −70°C before being used for RNA extraction.

### RNA purification and cDNA synthesis

Tissues were homogenized via TRIzol extraction (TRIzol reagent, Invitrogen, USA) based on outlined instructions to extract the total RNA. The total RNA was treated with RNase-free DNase I (Promega, USA) for the removal of any contaminating DNA. RNA quality and concentration levels were determined using a photometer (Eppendorf Biophotometer, Germany), and RNA integrity was verified via electrophoresis. cDNA was synthesized with equal amounts of RNA samples using an oligo (deoxythymidine) 15 primer and M-MLV reverse transcriptase (TaKaRa, Japan) based on manufacturer instructions.

### Gene cloning and sequence analysis

Specific PCR primers were selected using oligo 7.0 and primer premier 5.0. Pig NMB and NMBR genes were amplified from cDNA by PCR, using primers based on sequences of NMB and NMBR genes (GenBank ID: NM_001123145.1 and NM_001128489.1). Sequences of the PCR primers are shown in Table 1 [7]. PCR amplification products were extracted using a Gel/PCR DNA fragments extraction kit (Geneaid, Taiwan) and were ligated into the pUM 19-T vector (Vazyme Biotech Co., Ltd, China). The recombinant plasmid was transformed into Escherichia coli. The clones were sequenced entirely using vector primers to identify inserted regions (Invitrogen, Shanghai, China), and sequences were then submitted to GenBank.

**Table 1:** Nucleotide sequence of PCR primers and PCR conditions.

Genes	Primer sequence (5'-3')	PCR conditions	Length (bp)
NMB	Forward CATGACCCTGCGGGCTAGG	94°C 5min, (94°C 30s, 58°C 30s, 72°C 30 s) 40 cycles, 72°C 7min	567
	Revers GAAGCAAGACATACAGCAGGGACG		
NMBR	Forward ATGCCCCCCAAGTCTCTTTC	94°C 5min, (94°C 30s, 55°C 30s, 72°C 30 s) 40 cycles, 72°C 7 min	1173
	Revers TCACAGTGCCATTTCCTGTTTC		

NCBI BLAST was utilized to perform the identity/similarity assessment and the homology search (http://www.ncbi.nlm.nih.gov/blast). The protein domain determination and characterization were performed using the Bioedit software and SMART (http://smart.emblheidelberg.de/). The phylogenetic analysis was performed using the neighbor joining method in MEGA 4.0, and the alignment was performed using the ClustalW algorithm. TMHMM (http://www.cbs.dtu.dk/services/TMHMM-2.0) was used to predict the transmembrane domains.

### Relative real-time RT-PCR

An Applied Biosystens 7500 sequence detector (Applied Biosystems, CA) was used to perform relative real-time PCRs using the SYBR^®^ Green Master Mix Kit (Vazyme Biotech Co., Ltd, China). Reaction protocols followed the following format: 5 min at 95°C for enzyme activation followed by 40 cycles of 10 s at 95°C, 34 s at the appropriate annealing temperature, 15 s at 95°C, 60 s at 60°C, and 15 s at 95°C. Amplifications were conducted in duplicate using the gene-specific primers shown in Table 2, and the housekeeping gene ß-actin (GenBank ID: XM 003124280.2) was used as an internal control. Fluorescence was monitored during each PCR cycle at the annealing stage. A melting-curve analysis was conducted to determine the specificity of the amplification at the end of the PCR, and product purity levels were examined via electrophoresis and sequencing.

**Table 2:** Primers and annealing temperature for real-time PCR.

Genes	Primer sequence (5'-3')	Annealing (°C)	Length (bp)
NMB	Forward CTACTGGGGACAACGCACCAC	60	146
	Reverse ACCAGCAGCCTCCTGTACGG		
NMBR	Forward TAGGCCACATGATTGTCAC	60	136
	Reverse GACTTCCTTCCGCAACAGA		
β-actin	Forward CTCCATCATGAAGTGCGACGT	60	114
	Reverse GTGATCTCCTTCTGCATCCTGTC		

The NMB and NMBR mRNA quantities were expressed as a proportion of the ß-actin mRNA quantity using the 2^-ΔΔCT^ method [38], where ΔC_T_ was obtained by subtracting the corresponding ß-actin C_T_ value from the specific C_T_ of the target (NMB or NMBR), and ΔΔC_T_ was determined by subtracting the ΔC_T_ of each experimental sample from ΔC_T_ of the reference sample (the tissue with the lowest expression).

### Immunohistochemistry

Tissues were sectioned (5 μm thick) using a Rotary Microtome YD-1508R (Jinhua YIDI Medical Appliance CO., Ltd, China), deparaffinized in xylene and then dehydrated in graded series of ethanol.

The avidin-biotin-peroxidase complex (ABC) method was used to perform the Immunohistochemistry procedure. Sections were pretreated with 0.1 M PBS (5 min). The endogenous peroxidases were quenched with methanol containing 0.3% hydrogen peroxide (10 min) and tissue was then rinsed with 0.1 M PBS (10 min). The sections were blocked with 5% normal rabbit serum (30 min), incubated in primary antisera at 4°C overnight (goat anti-NMBR polyclonal antibody, GeneTex diluted 1:50 in 0.1 M PBS, Santa Cruz Biotechnology, Inc., USA), and then rinsed in 0.1 M PBS. The sections were then incubated in biotinylated rabbit-anti-goat IgG (Wuhan Boster Biological Technology Co., Ltd, China) for 30 min, were rinsed with PBS after being incubated in streptavidin-biotin complex (Wuhan Boster Biological Technology Co., Ltd, China) for 30 min, and were then washed again. Sections were stained using a freshly prepared 0.02% DAB (Wuhan Boster Biological Technology Co., Ltd, China) and were counterstained with hematoxylin. Images of tissue sections for the NMBR immunohistochemistry procedure were acquired using an OLYMPUS BX43 microscope (Olympus Corporation, Tokyo, Japan) to determine the localization of NMBR immunoreactive cells. Negative control tests were conducted via primary antisera pre-absorption using its respective antigen; normal rabbit serum (Wuhan Boster Biological Technology Co., Ltd, China) was used rather than the primary antibody.

### Statistical analysis

All data are shown as means ± SEM. P < 0.05 deems a significant difference. The statistical analysis was carried out using one-way ANOVA and the independent sample t-test with SPSS Statistics 19.0.

## Results

### Gene cloning and sequence analysis

**Fig 1:**
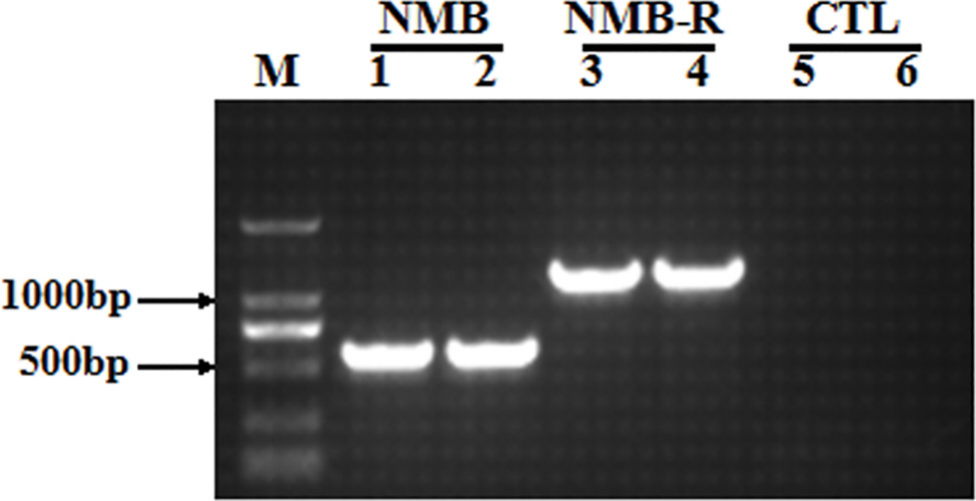
Gel electrophoresis of pig NMB and NMBR PCR products. Lane 1, 2: NMB; Lane 3, 4: NMBR; M: DNA Marker DL 2000; Lane 5, 6: negative control obtained without the addition of cDNA.

**Fig 2:**
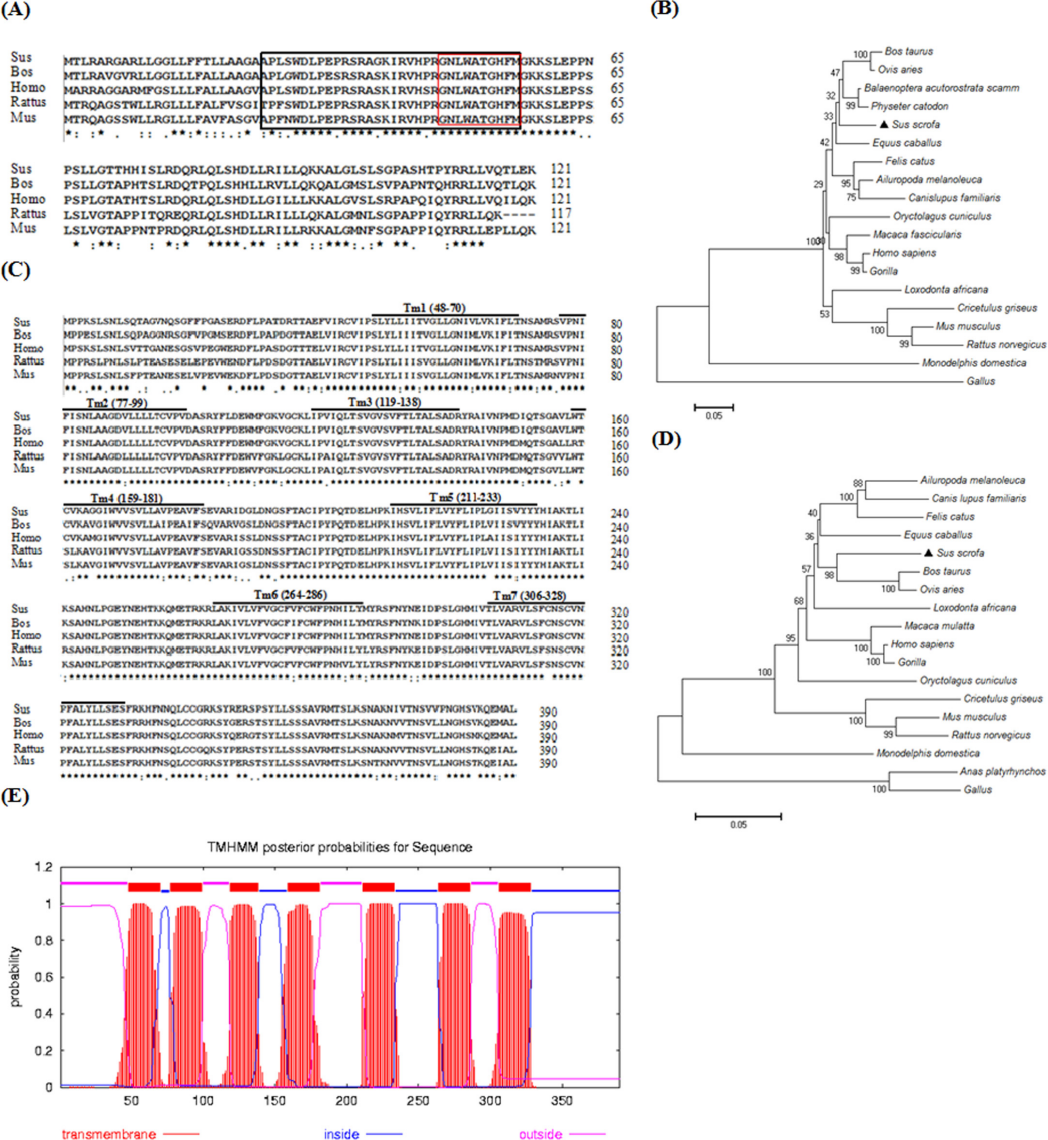
Sequence analysis of the pig NMB precursor and NMBR. Panel (A): multiple alignment of deduced aa sequences of the pig NMB precursor in several mammals. The conserved sequence of NMB-32 and NMB-10 is shown in the box. GenBank ID: *Bos taurus* NP_001068738.1, *Homo sapiens* AAH08603.1, *Rattus norvegicus* NP_001102619.1, and *Mus musculus* NP_080799.1. Identifiers: “*” identical, “.” and “:” conservative replacements, “” mismatches. Panel (B): phylogenetic tree analysis of NMB aa sequences. Panel (C): multiple alignment of the deduced aa sequences of the pig NMBR in several mammals. The putative transmembrane regions are indicated by horizontal bars. GenBank ID: *Bos taurus* NP_001192639.1, *Homo sapiens* AAA59939.1, *Rattus norvegicus* NP_036931.1, and *Mus musculus* NP_032729.1. Panel (D): phylogenetic tree analysis of NMBR aa sequences. Panel (E): transmembrane region of the pig NMBR aa sequence fragment. 1–47 aa, 100–118 aa, 182–210 and 287–305 aa, outside sequence; 48–70 aa, 77–99 aa, 119–138 aa, 159–181 aa, 211–233 aa, 264–286 aa and 306–328 aa, TMhelix sequence; 71–76 aa, 139–158 aa, 234–263 aa and 329–390 aa, inside sequence.

We cloned pig NMB and NMBR gene fragments via reverse transcription PCR (RT-PCR), and final PCR product size of 567 and 1,173 bp were found, respectively (Fig 1). The DNA sequencing and bioinformatic analysis results show that pig NMB has one open reading frame (ORF) of 366 bp and encodes 121 amino acids (which includes the mature peptide of NMB-32 (APLSWDLPEPRSRAGKIRVHPRGNLWATGHFM)) and that the main protein structure of prepro-NMB presents high levels of homology with other animal species (Fig 2A). Furthermore, NMBR contains one ORF of 1,173 bp and encodes 390 amino acids (which include typical transmembrane domain features (Fig 2E)), and the transmembrane domains of NMBR (Fig 2C) are fairly conserved in pigs, humans, mice, rats and cows. PCR products were confirmed for NMB and NMBR genes via sequencing, and the NMBR sequence was submitted to GenBank (GenBank ID: KM058699).

Furthermore, nucleotide sequences and amino acids sequences of the NMB and its receptor were analyzed using the bioinformatics software. The results show that the NMB nucleotide sequences and amino acids sequences are highly homologous to those of the *Balaenoptera* (90% and 86%) and *Physeter catodon* (89% and 86%) (Table 3); the NMBR nucleotide sequences and amino-acids sequences are highly homologous to those of the *Bos taurus* (91% and 92%) and *Ovis aries* (91% and 92%) (Table 4). Phylogenetic analyses show that the NMB gene sequences remain largely conserved in other vertebrates (Fig 2B) and that NMBR coding sequences are clustered with other species (Fig 2D).

**Table 3:** Comparision of NMB nucleotide sequences and amino acids between the pig and other species.

Species	GenBank ID	Identity percentage (%)	
		Nucleotide	Amino acid
*Balaenoptera*	XM_007194296.1/XP_007194358.1	90%	86%
*Physeter catodon*	XM_007105771.1/XP_007105833.1	89%	86%
*Oryctolagus cuniculus*	XM_002721480.2/XP_002721526.1	87%	79%
*Ovis aries*	XM_004017766.1/XP_004017815.1	86%	83%
*Equus caballus*	XM_005602767.1/XP_001502657.3	86%	83%
*Loxodonta africana*	XM_003413872.1/XP_003413920.1	85%	77%
*Bos taurus*	NM_001075270.2/NP_001068738.1	85%	81%
*Macaca fascicularis*	XM_005560395.1/XP_005560452.1	85%	80%
*Homo sapiens*	NM_021077.3/AAH08603.1	84%	78%
*Ailuropoda melanoleuca*	XM_002919807.1/XP_002919853.1	84%	84%
*Felis catus*	XM_003986824.2/XP_003986873.1	84%	82%
*Gorilla*	XM_004056696.1/XP_004056743.1	84%	79%
*Canis lupus familiaris*	XM_536200.4/XP_536200.1	82%	71%
*Cricetulus griseus*	XM_007621840.1/XP_007620031.1	79%	72%
*Gallus gallus*	NM_001079476.2/NP_001072944.1	79%	41%
*Mus musculus*	NM_001291280.1/NP_080799.1	77%	74%
*Rattus norvegicus*	NM_001109149.1/NP_001102619.1	74%	75%
*Monodelphis domestica*	XM_001364350.2/XP_007620031.1	73%	49%

**Table 4:** Comparision of NMBR nucleotide sequences and amino acids between the pig and other species.

Species	GenBank ID	Identity percentage (%)	
		Nucleotide	Amino acid
*Bos taurus*	NM_001205710.1/NP_001192639.1	91%	92%
*Ovis aries*	XM_004011382.1/XP_004011431.1	91%	92%
*Equus caballus*	XM_001502752.3/XP_001502802.3	90%	91%
*Macaca mulatta*	NM_001278400.1/NP_001265329.1	89%	90%
*Homo sapiens*	NM_002511.2/AAA59939.1	89%	90%
*Ailuropoda melanoleuca*	XM_002912637.1/XP_002912683.1	89%	89%
*Felis catus*	XM_003986612.2/XP_003986661.1	89%	89%
*Gorilla*	XM_004044764.1/XP_004044812.1	89%	90%
*Canis lupus familiaris*	XM_849337.3/XP_854430.2	88%	89%
*Loxodonta africana*	XM_003403986.1/XP_003404034.1	88%	89%
*Oryctolagus cuniculus*	XM_002714848.2/XP_002714894.2	87%	87%
*Mus musculus*	NM_008703.2/NP_032729.1	84%	87%
*Rattus norvegicus*	NM_012799.1/NP_036931.1	84%	86%
*Cricetulus griseus*	XM_003501188.1/ERE85002.1	84%	87%
*Monodelphis domestica*	XM_001380878.2/XP_001380915.1	80%	84%
*Anas platyrhynchos*	XM_005010018.1/EOB08326.1	77%	79%
*Gallus gallus*	XM_426167.3/XP_426167.1	77%	78%

### NMB and NMBR mRNA expression in various pig tissues

**Fig 3:**
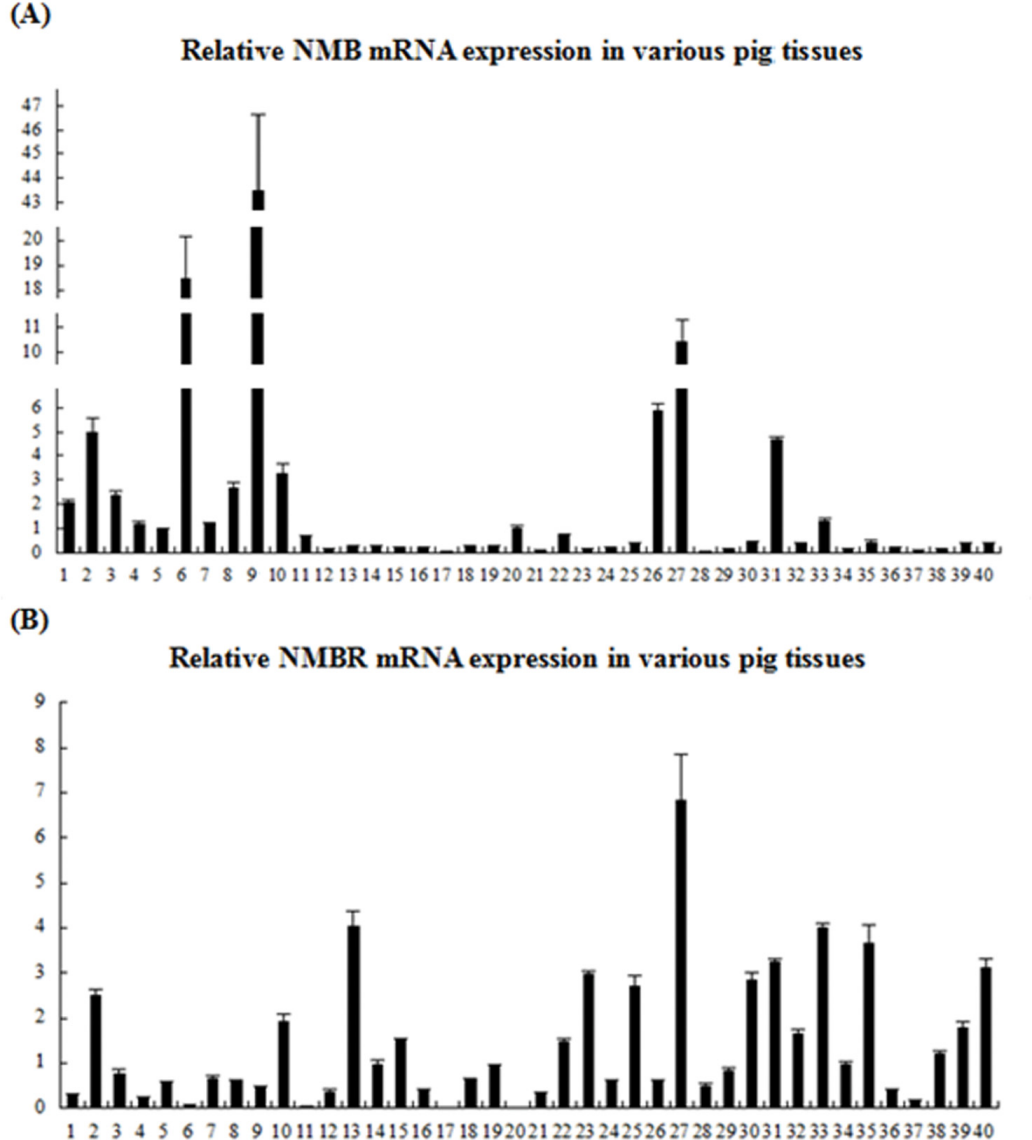
Relative NMB and NMBR mRNA levels in the 40 pig tissues. 1.cerebral cortex; 2.cerebellum; 3.spinal cord; 4.medulla oblongata; 5.pons; 6.midbrain; 7.hypothalamus; 8.hippocampus; 9.olfactory bulb; 10.hypophysis; 11.heart; 12.liver; 13.spleen; 14.lung; 15.kidney; 16.stomach; 17.duodenum; 18.jejunum; 19.ileum; 20.cecum; 21.colon; 22.rectum; 23.parotid gland; 24.mandibular gland; 25.thymus; 26.adrenal gland; 27.pancreas; 28.bone marrow; 29.pharyngeal tonsil; 30.jejunum lymph nodes; 31.testis; 32.epididymis; 33.ovary; 34.uterus; 35.esophagus; 36.trachea; 37.aorta; 38.bladder; 39.fat; 40.muscle. The 2^-△△CT^ method is used to calculate the relative NMB (A) and NMBR (B) mRNA expression levels. Values are the mean ± SEM of three samples; one sample per pig.

Relative expression levels of NMB and NMBR mRNA were detected in all 40 tissues through real-time PCR. Specificity reactions were confirmed via melting curve analyses so that NMB, NMBR and β-actin products were shown as a single PCR product. The total RNA of each tissue was reverse transcribed cDNA under conditions of reverse transcriptase, and the CT values did not differ from those of the blanks (>30 cycles), indicating that genomic DNA does not contribute to NMB and NMBR mRNA quantities. These results are shown in (Fig 3).

The results show that NMB mRNA expression in the central nervous system is more pronounced than that in peripheral tissues. The highest levels of NMB mRNA were found in the olfactory bulb. Relatively high levels of NMB mRNA were mostly found in the CNS (e.g., the cerebral cortex, cerebellum, spinal cord, medulla oblongata, midbrain, hypothalamus, hippocampus, and hypophysis) and in peripheral tissues such as the pancreas, adrenal gland, testis, ovary and cecum. Moderate levels of NMB mRNA were found in the rectum, heart and pons, with low expression levels detected in the bone marrow and duodenum. Either absent or very low NMB mRNA expression levels were found in other tissues.

However, relatively high expression levels of NMBR mRNA were detected in the peripheral tissues, which differ from NMB. The highest levels of NMBR mRNA were found in the pancreas, and high levels of NMBR mRNA were mainly found in the spleen, ovary, esophagus, testis, muscles, parotid gland, jejunum lymph nodes, thymus, fat, epididymis, kidneys, rectum, bladder, cerebellum and hypophysis of the CNS. Moderate levels of NMBR mRNA were found in the uterus, lungs, ileum, tonsil, jejunum, mandibular gland, adrenal gland, bone marrow, spinal cord, hypothalamus, hippocampus and pons. Other tissues showed no or low levels of expression.

### Relative levels of NMB and NMBR expression during the sow estrous cycle

**Fig 4:**
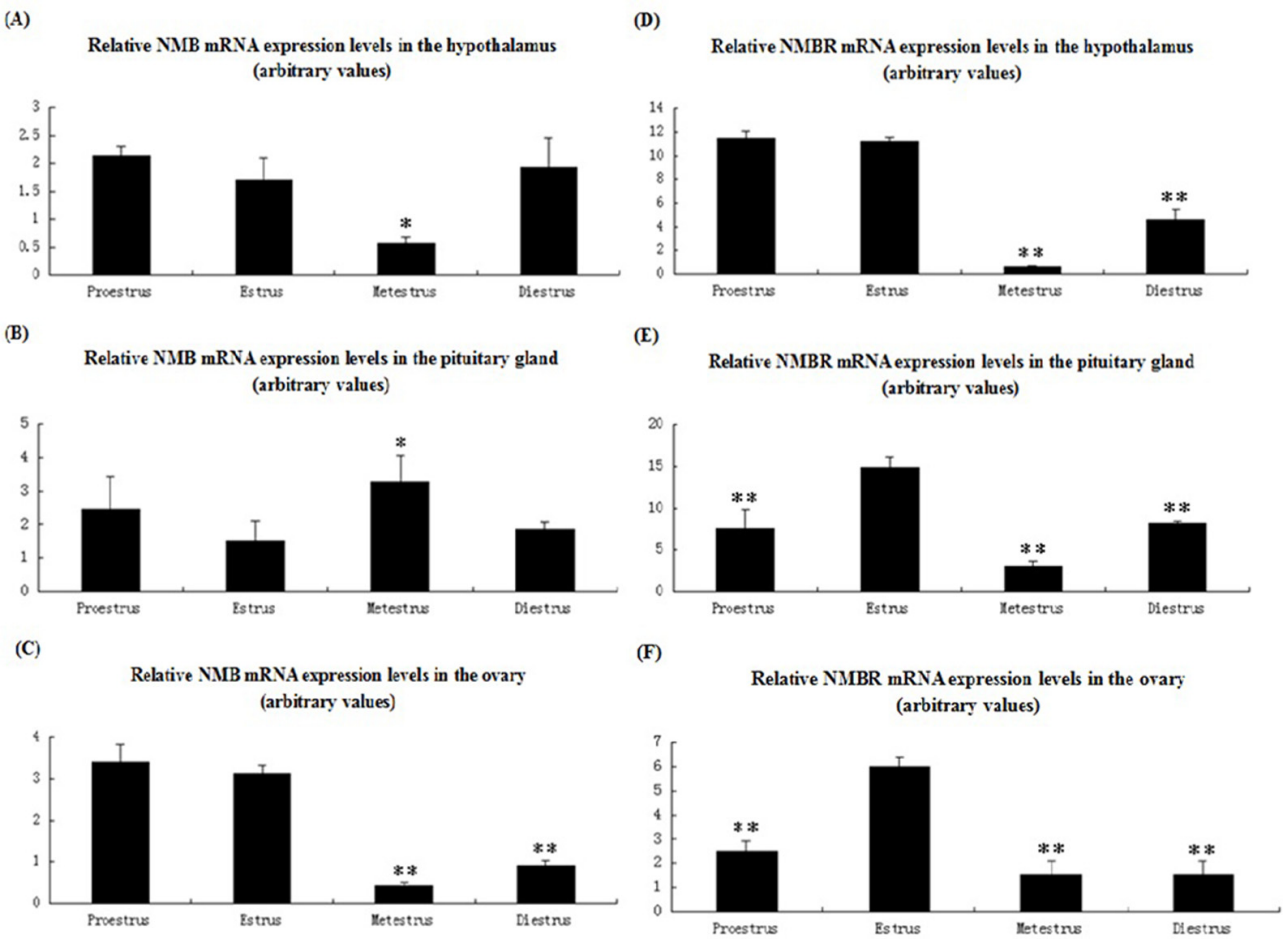
NMB and NMBR mRNA expression pattern in the reproductive axis throughout the sow estrous cycle. NMB and NMBR expression patterns in the hypothalamus (A and D), pituitary (B and E), and ovaries (C and F) are shown. Relative levels of NMB and NMBR mRNA were calculated as shown in Fig 3. Values are the mean ± SEM values of three pigs per phase group. Relative to the estrus group, an asterisk denotes a significant difference. *P < 0.05; **P < 0.01.

Relative expression levels of NMB and NMBR mRNA were detected in the hypothalamus-pituitary-ovary (HPO) axis of the sow estrous cycle, as shown in (Fig 4).

In the hypothalamus (Fig 4A and 4D), NMB and NMBR mRNA level patterns were similar. The highest levels of NMB and NMBR expression were found during proestrus (though in relation to the estrus group, these levels were not significant), whereas the lowest expression levels were found during metestrus (*P* < 0.05 and *P* < 0.01). During diestrus, hypothalamic NMB expression levels returned to estrus levels and slightly exceeded estrus levels. Although NMBR expression levels during diestrus were higher than metestrus levels, they declined significantly (*P* < 0.01).

In the pituitary (Fig 4B and 4E), the lowest relative amounts of NMB mRNA were found during estrus, whereas peak levels were measured during metestrus (*P* < 0.05). From diestrus to proestrus, pituitary NMB expression increased. However, an inverse expression pattern was found in the pituitary NMBR mRNA as the highest NMBR expression levels were observed during estrus. Although the lowest levels of NMBR expression were detected during metestrus (*P* < 0.01), pituitary NMBR mRNA levels gradually declined from diestrus to proestrus.

In the ovaries (Fig 4C and 4F), peak NMB expression levels were observed during proestrus, which were not significant compared with estrus levels. The lowest expression levels were measured during metestrus (*P* < 0.01). During diestrus, NMB mRNA levels were higher than metestrus levels but significantly decreased (*P* < 0.01) relative to estrus levels. However, the highest NMBR expression levels occurred during estrus, whereas the lowest levels of NMBR expression were detected during metestrus (*P* < 0.01). During the proestrus and diestrus phases, NMBR expression levels significantly decreased (*P* < 0.01 and *P* < 0.01).

### Relative levels of NMB and NMBR expression during boar post-natal development

**Fig 5:**
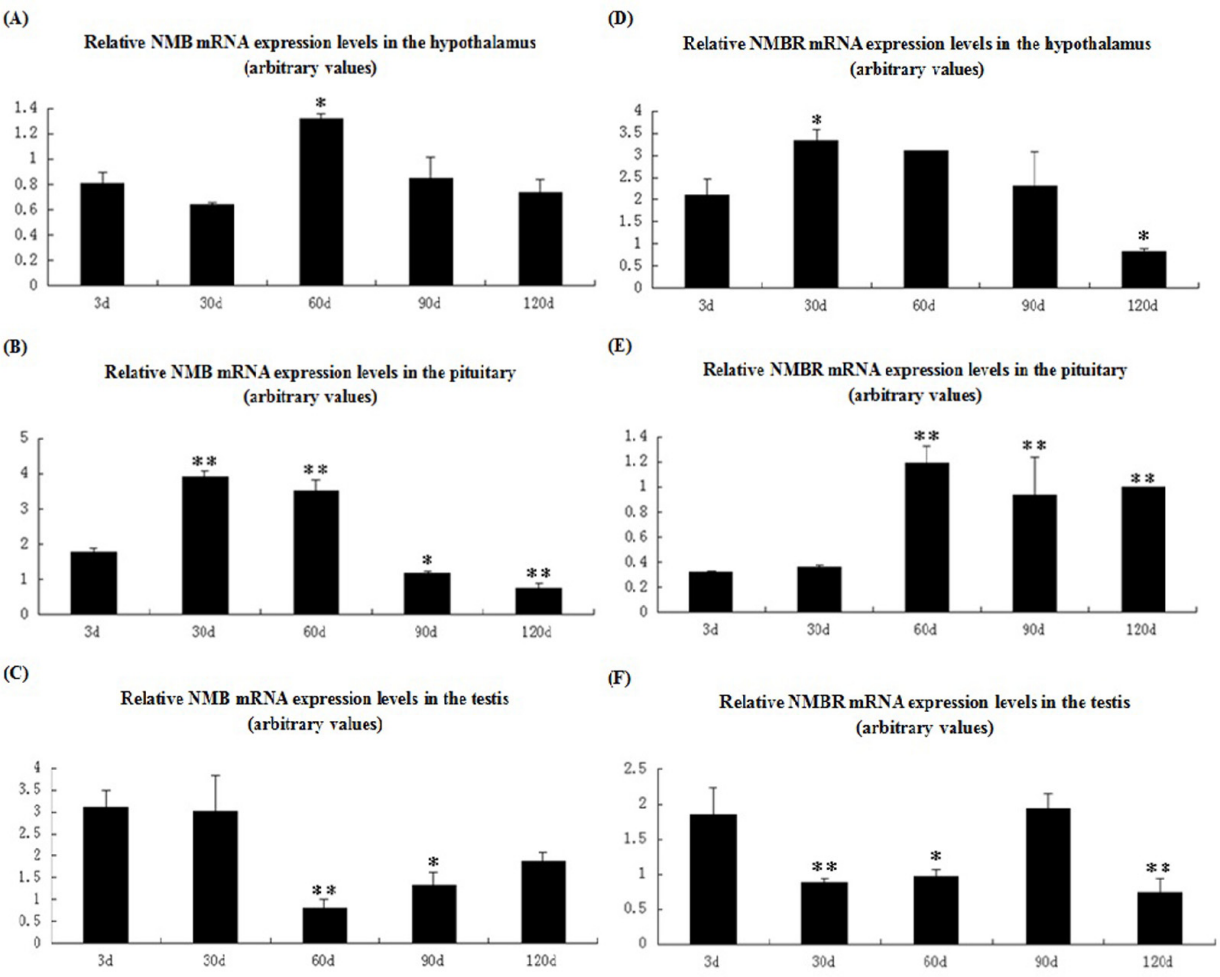
NMB and NMBR mRNA expression patterns alone the reproductive axis over the course of male pig sexual development. NMB and NMBR expression patterns in the hypothalamus (A and D), pituitary (B and E), and testis (C and F) are shown. Relative levels of NMB and NMBR mRNA were calculated as shown in Fig 3. Values are mean ± SEM values for three pigs per phase group. Relative to the 3d group, an asterisk denotes a significant difference. *P < 0.05; **P < 0.01.

Relative expression levels of NMB and NMBR mRNA were investigates in the hypothalamus-pituitary-testicular (HPT) axis of boar post-natal development and are shown in (Fig 5).

In the hypothalamus (Fig 5A and 5D), peak expression levels of NMB mRNA were observed on day 60 (*P* < 0.05). Although the lowest levels of NMB expression were detected on day 30, changes in hypothalamic NMB mRNA levels found on days 30, 90 and 120 were not significant. NMBR mRNA expression reached peak levels on day 30 (*P* < 0.05), followed by a gradual decrease from days 30 to 120. The lowest NMBR expression levels were detected on day 120 (*P* < 0.01).

In the pituitary gland (Fig 5B and 5E), the highest expression level of NMB occurred on day 30 (*P* < 0.01), whereas the lowest level was observed on day 120 (*P* < 0.01). At the same time, relative to levels found on day 3 of pituitary NMB mRNA expression, we found a significant increase on day 60 (*P* < 0.01) and a significant decrease on day 90 (*P* < 0.01). However, the lowest expression of NMBR mRNA was measured on day 3. On day 30, the pituitary NMBR expression level was slightly higher than that for day 3. Peak NMBR expression levels were detected on day 60 (*P* < 0.01), changes in pituitary NMBR mRNA levels on days 90 and 120 increased significantly.

In the testis (Fig 5C and 5F), peak levels of NMB mRNA were found on day 3, whereas the lowest levels were detected on day 60 (*P* < 0.01). On day 30, NMB expression levels were slightly lower than those detected on day 3. NMB expression levels then gradually increased from day 60 to day 120. Day 3 NMBR expression levels were higher than those of day 120. Peak NMBR expression levels occurred on day 90 and were slightly higher than day 3 levels. The lowest expression of NMBR mRNA occurred on day 120 (*P* < 0.01), and levels significantly decreased (*P* < 0.01 and *P* < 0.05) on days 30 and 60 relative to day 3 levels.

### NMBR protein localization in various pig tissues

NMBR protein distributions were detected in peripheral pig tissues via immunohistochemistry (IHC) and were found to be widely expressed in a variety of tissues and organs.

**Fig 6:**
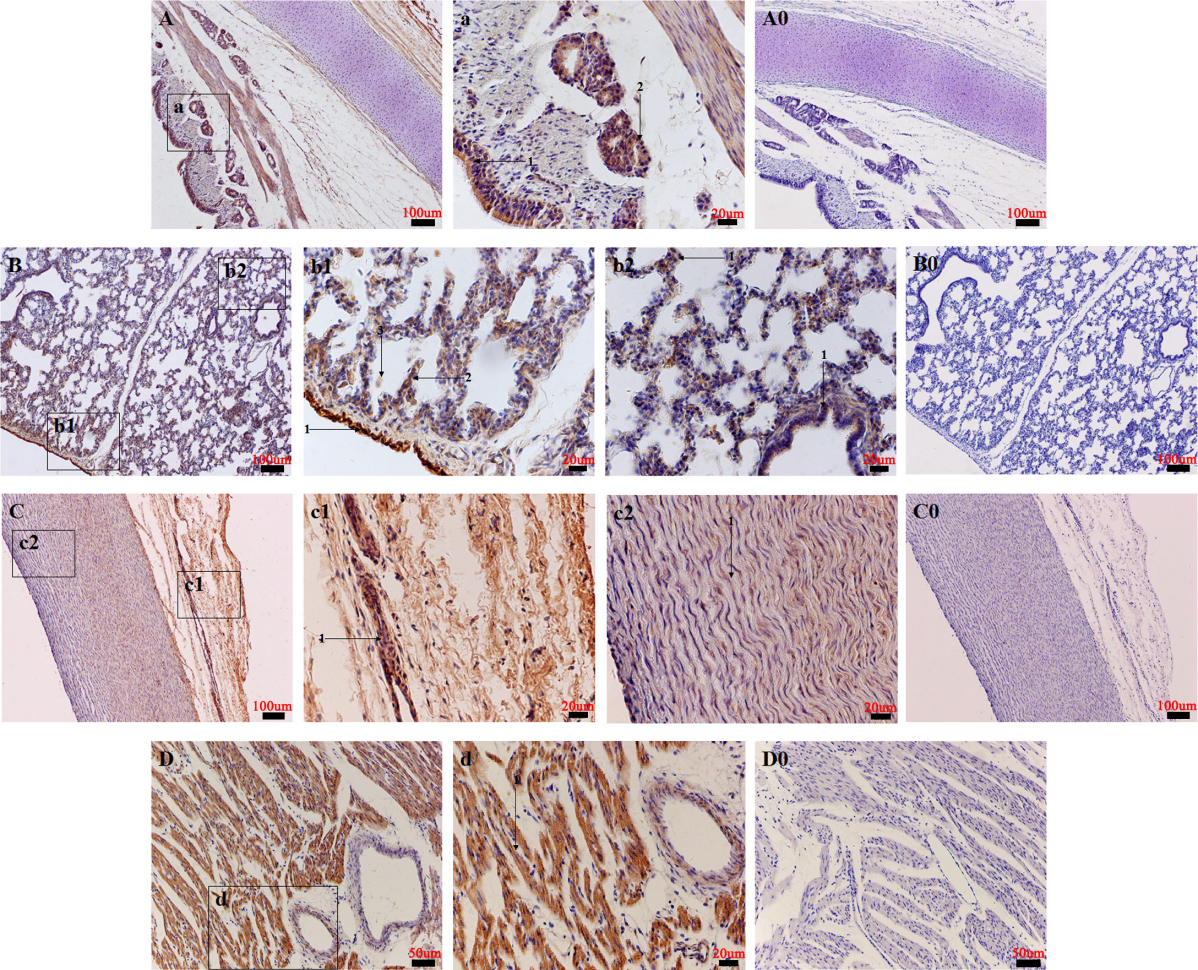
Distribution of NMBR in pig respiratory system and circulatory system. Immunohistochemical staining of NMBR in the trachea (A), lungs (B), aorta (C) and heart (D). High NMBR-positive cell magnification in the pseudostratified ciliated columnar epithelium of the trachea (1) (denoted by an arrow) and in tracheal glands of the tracheal submucosa (2) (denoted by an arrow) (a); the visceral pleura (1) (denoted by an arrow), the alveolar epithelium cells (2) (denoted by an arrow), the pulmonary alveolar macrophage (3) (denoted by an arrow) (b1), and the simple columnar ciliated epithelium of the terminal bronchiole (1) (denoted by an arrow) (b2); the nerve (1) (denoted by an arrow) (c1) and elastic fibers of tunica media in the aorta (1) (denoted by an arrow) (c2); cardiac cells (1) (denoted by an arrow) in the heart (d). Negative controls in the trachea (A0), lungs (B0), aorta (C0) and heart (D0). Scale bars in (A, B, C, A0, B0, and C0) = 100 μm, (D and D0) = 50 μm, (a, b1, b2, c1, c2 and d) = 20 μm.

Respiratory system: NMBR staining was found in the pseudostratified ciliated columnar epithelium, in tracheal glands of the submucosa and in the muscularis of the trachea (Fig 6A). In the lungs, NMBR staining was found in the visceral pleura, in alveolar epithelium cells, in the pulmonary alveolar macrophage, in the simple cuboidal ciliated epithelium of the respiratory bronchiole and in the simple columnar ciliated epithelium of the terminal bronchiole (Fig 6B).

Circulatory system: In the aorta, NMBR immunoreactivity was found in elastic fibers of the tunica media and nerve (Fig 6C). In the heart, the NMBR was found concentrated in the cardiac cells (Fig 6D).

**Fig 7:**
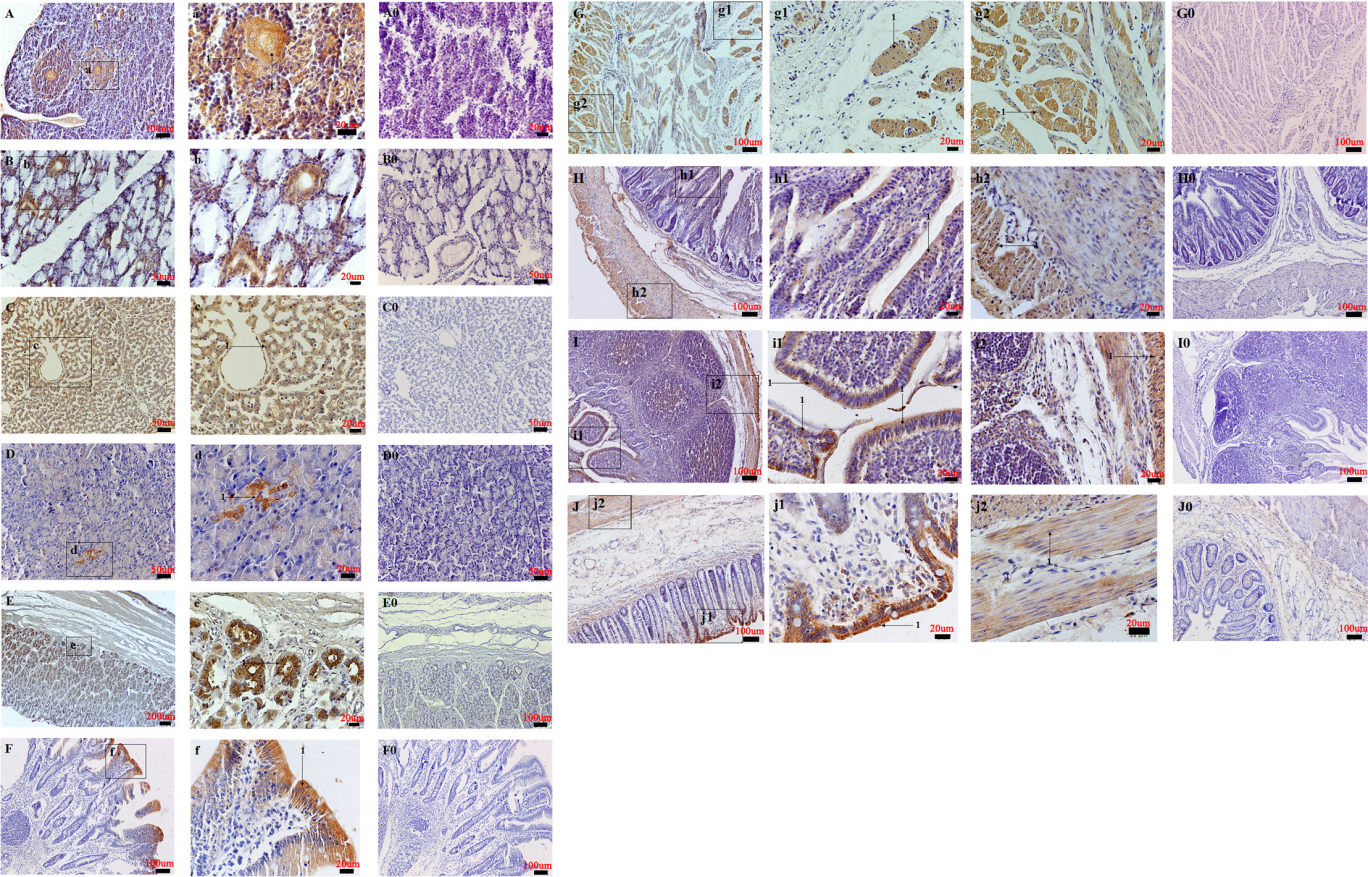
Distribution of NMBR in the pig digestive system. The NMBR immunostaining results in the parotid gland (A), submandibular gland (B), liver (C), pancreas (D), esophagus (G), stomach (E), duodenum (F), jejunum (H), ileum (I) and rectum (J). High magnification of NMBR-positive cells in the intralobular ducts (1) (denoted by an arrow) (a); the ducts (b); the hepatocytes (1) (denoted by an arrow) and the central vein (c); the pancreatic islets (1) (denoted by an arrow) (d); the mucous glands (1) (denoted by an arrow) (g1) and the skeletal muscle of the muscular layer of the esophagus (1) (denoted by an arrow) (g2); the chief cell and parietal cell of the fundic gland (1) (denoted by an arrow) (e); the absorptive cell and lymphocyte of the intestinal villi (1) (denoted by an arrow) (f); the villus (1) (denoted by an arrow) (h1 and i1) and muscular layer (1) (denoted by an arrow) (h2 and i2) in the jejunum and ileum; the simple columnar epithelium (1) (denoted by an arrow) (j1) and muscular layer (1) (denoted by an arrow) (j2) in the rectum. Negative controls in the parotid gland (A0), submandibular gland (B0), liver (C0), pancreas (D0), esophagus (G0), stomach (E0), duodenum (F0), jejunum (H0), ileum (I0) and rectum (J0). Scale bars in (E) = 200 μm, (A, F, G, H, I, J, E0, F0, G0, H0, I0, and J0) = 100 μm, (B, C, D, B0, C0 and D0) = 50 μm, (a, b, c, d, e, f, g1, g2, h1, h2, i1, i2, j1 and j2) = 20 μm.

Digestive system: In the parotid gland, NMBR staining was found in the intralobular ducts (Fig 7A). Analogously, in the submandibular gland, NMBR immunoreactivity found seen in the ducts (Fig 7B). In the liver, the NMBR was widely expressed in hepatocytes and in the central vein (Fig 7C). In the pancreas, the NMBR was found concentrated in pancreatic islets (Fig 7D). Mucous glands and skeletal muscles of the muscular layer of the esophagus were found to be highly immunoreactive (Fig 7G). In the stomach, the NMBR was strongly expressed in chief and parietal cells of the fundic gland (Fig 7E). In the duodenum, NMBR immunoreactive cells were found in absorptive cells and lymphocytes of intestinal villi (Fig 7F). In the jejunum and ileum, NMBR staining was detected in the villus and muscular layers (Fig 7H and 7I). In the large intestine, the cecum and colon did not show any indications of NMBR staining, whereas high levels of immunoreactivity were found in the simple columnar epithelium and muscular layers of the rectum (Fig 7J).

**Fig 8:**
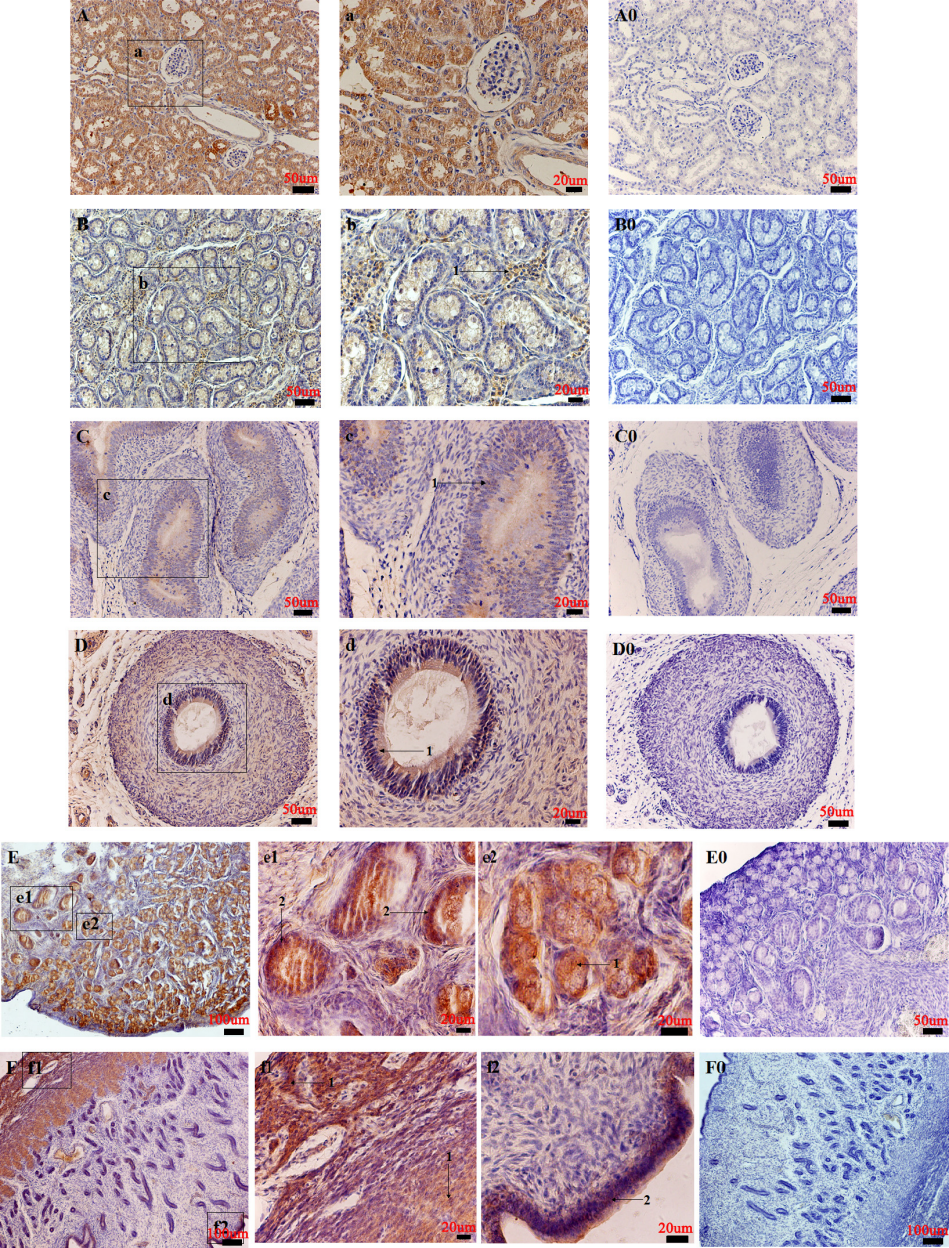
Distribution of NMBR in the pig urogenital system. The NMBR immunostaining results in the kidney (A), testis (B), epididymis (C), ductus deferens (D), ovary (E), uterus (F). High magnification of NMBR-positive cells in the proximal tubule and the distal tubule of the renal cortex (a); the leydig cells (1) (denoted by an arrow) (b); the pseudostratified columnar epithelium of the epididymal duct (1) (denoted by an arrow) (c); the pseudostratified epithelium (1) (denoted by an arrow) and muscular layer of the ductus deferens (d); the primary follicle, the secondary follicle (1) (denoted by an arrow) and the granulosa cells (2) (denoted by an arrow) (e1 and e2); the myometrium (1) (denoted by an arrow) (f1) and endometrium (2) (denoted by an arrow) (f2). Negative controls in the kidney (A0), testis (B0), epididymis (C0), ductus deferens (D0), ovary (E0), uterus (F0). Scale bars in (E, F and F0) = 100 μm, (A, B, C, D, A0, B0, C0, D0 and E0) = 50 μm, (a, b, c, d, e1, e2, f1 and f2) = 20 μm.

Urogenital system: In the kidneys, the NMBR was found highly concentrated in the proximal tubule and in the distal tubule of the renal cortex (Fig 8A). In the testis, high levels of NMBR immunoreactivity were found in leydig cells, whereas negative results were found for sertoli cells and spermatogonia of the seminiferous tubules (Fig 8B). In the epididymis, moderate NMBR immunoreactivity levels were found in the pseudostratified columnar epithelium of the epididymal duct (Fig 8C). In the ductus deferens, NMBR expression was detected in the pseudostratified epithelium and in the muscular layer of the ductus deferens (Fig 8D). In the ovaries, primordial follicle showed high levels of NMBR staining. NMBR staining levels were also found to be high in the primordial, primary and secondary follicles. NMBR immunoreactivity was observed in granulosa cells of the primary and secondary follicles (Fig 8E). In the uterus, the endometrium and myometrium showed intense immunoreactivity (Fig 8F).

**Fig 9:**
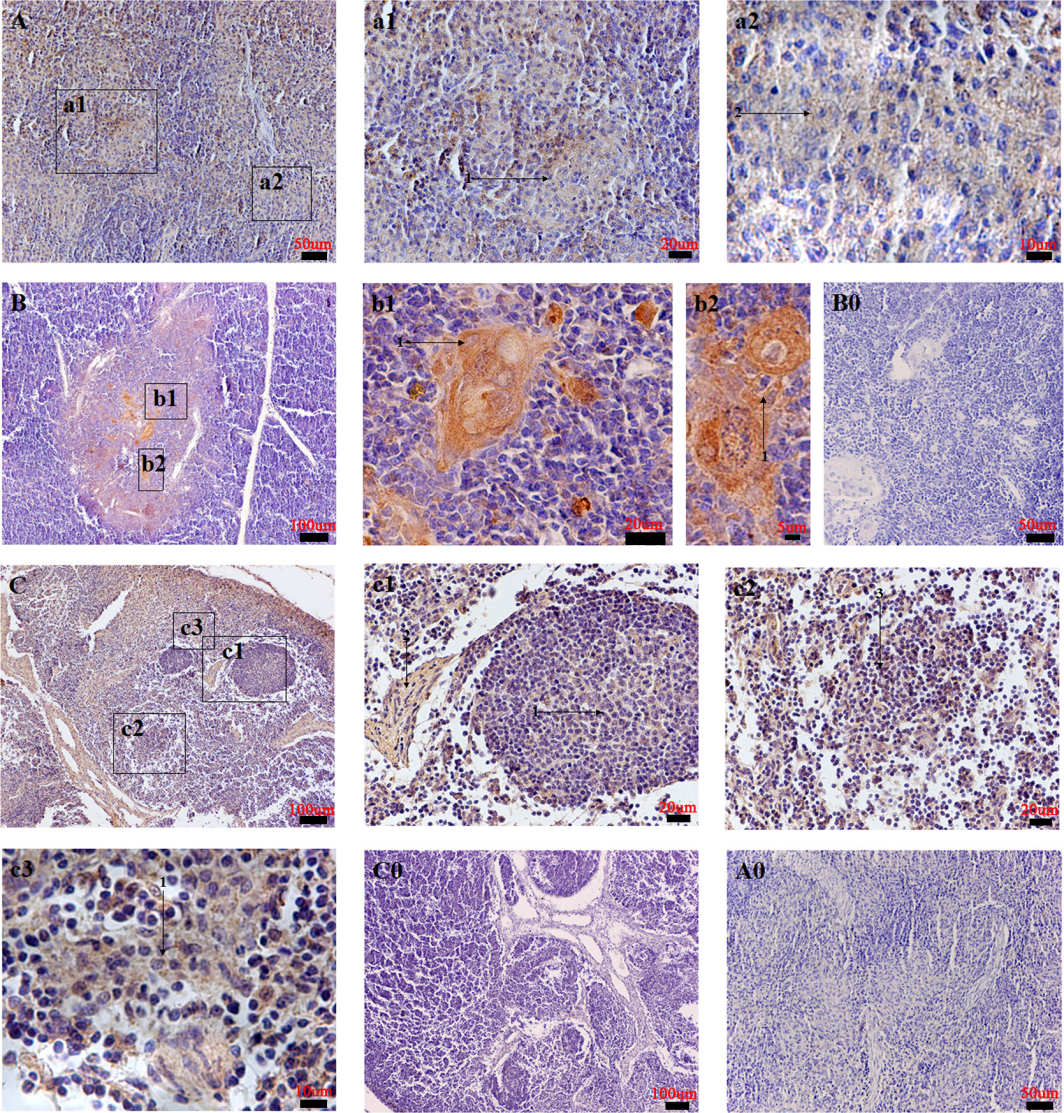
Distribution of NMBR in pig lymphatic organs. The NMBR immunostaining results in the spleen (A), the thymus (B) and the jejunum lymph node (C). High magnification of NMBR-positive cells in the periarterial lymphatic sheath (1) (denoted by an arrow), the trabecula and the ellipsoid (2) (denoted by an arrow) (a1 and a2); the thymic medulla and thymic corpuscle (1) (denoted by an arrow) (b1 and b2); the lymphoid nodule (1) (denoted by an arrow), the trabecula (2) (denoted by an arrow) (c1 and c3) and the diffuse lymphatic tissue (3) (denoted by an arrow) (c2). Negative controls in the spleen (A0), the thymus (B0) and the jejunum lymph node (C0). Scale bars in (B, C and C0) = 100 μm, (A, A0 and B0) = 50 μm, (a1, b1, c1 and c2) = 20 μm, (a2 and c3) = 10 μm, (b2) = 5 μm.

Lymphatic organs: In the spleen, NMBR immunoreactivity was diffusely observed in the periarterial lymphatic sheath, trabecula and ellipsoid (Fig 9A). In the thymus, NMBR staining was mainly found in the thymic medulla and thymic corpuscle (Fig 9B). In the jejunum lymph node, NMBR-positive cells were widely expressed in diffuse lymphatic tissues, the lymphoid nodule and the trabecula (Fig 9C).

**Fig 10:**
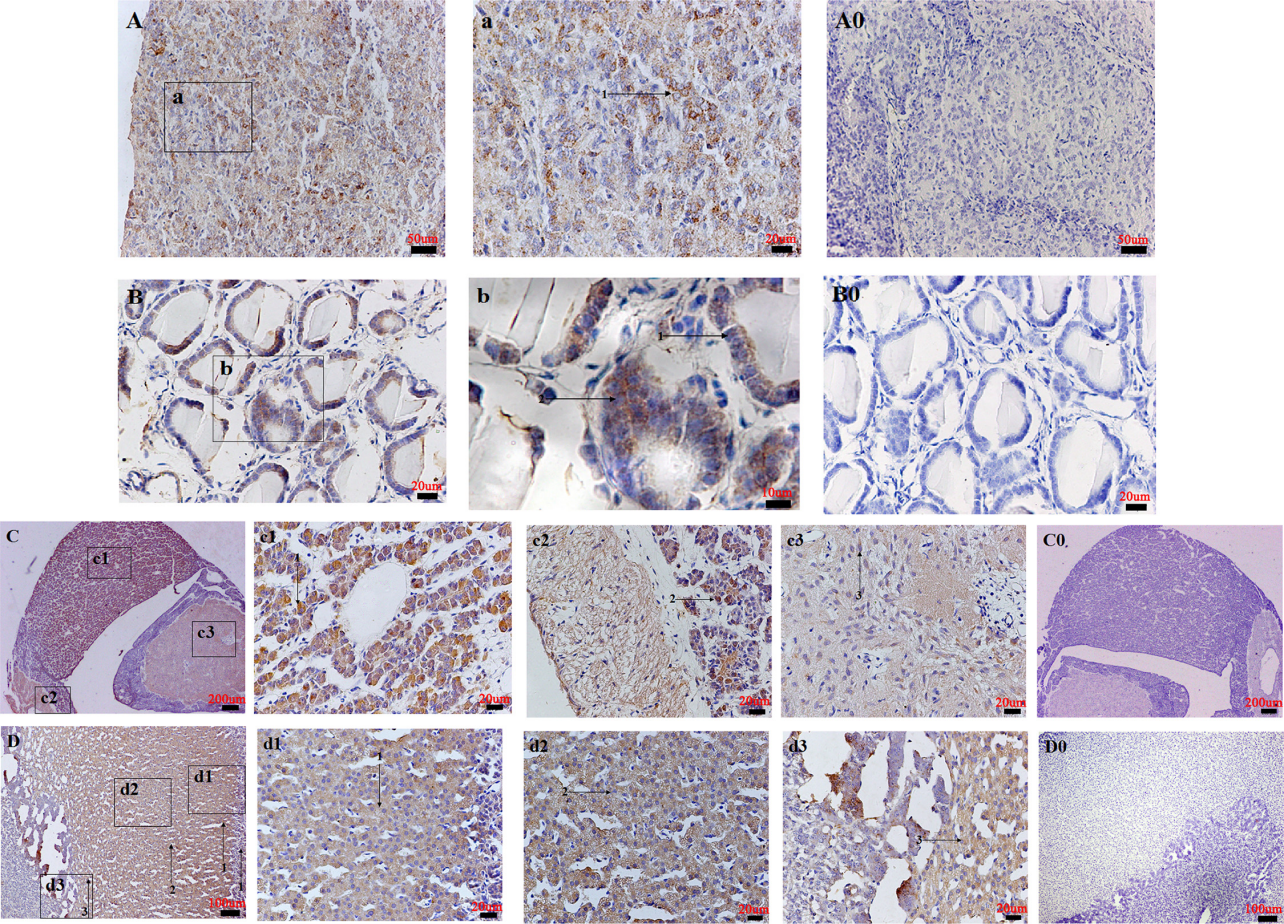
Distribution of NMBR in the pig endocrine system. The NMBR immunostaining results in the pineal body (A), the thyroid gland (B), the pituitary gland (C) and the adrenal gland (D). High magnification of NMBR-positive cells in the pinealocyte (1) (denoted by an arrow) (a); the follicular epithelial cells (1) (denoted by an arrow) and parafollicular cells (2) (denoted by an arrow) (b); the chromophil of the pars distalis (1) (denoted by an arrow) (c1), the pars intermedia (2) (denoted by an arrow), the neuroglial cell of the pars nervosa and pars tuberalis (3) (denoted by an arrow) (c2 and c3); the reticular zone (3) (denoted by an arrow) and fascicular zone of the adrenal cortex (1 and 2) (denoted by an arrow) (d1, d2 and d3). Negative controls in the pineal body (A0), the thyroid gland (B0), the pituitary gland (C0) and the adrenal gland (D0). Scale bars in (C and C0) = 200 μm, (D and D0) = 100 μm, (A and A0) = 50 μm, (B, B0, a, c1, c2, c3, d1, d2 and d3) = 20 μm, (b) = 10 μm.

Endocrine system: In the pituitary gland, NMBR immunoreactivity was widely observed in the chromophil of the pars distalis, in the pars intermedia, and in the neuroglial cell of the pars nervosa and pars tuberalis (Fig 10C). The pinealocyte of the pineal body showed moderate degrees of NMBR staining (Fig 10A). In the thyroid gland, NMBR-positive cells were detected in follicular epithelial and parafollicular cells (Fig 10B). In the adrenal gland, NMBR staining was found in reticular and fascicular zones of the adrenal cortex, whereas the adrenal medulla was found to be weakly immunostained (Fig 10D).

Negative controls based on normal rabbit serum showed no immunoreactivity results for NMBR.

## Discussion

The NMB/NMBR system, plays an important role in regulating physiological functions in humans and animals. However, gene sequences, distributions and physiological functions of NMB and NMBR in pigs remain unknown. Thus, in this experiment, we cloned pig NMB precursor and NMBR cDNA, detected expression levels of NMB and NMBR mRNA and distributions of NMBR proteins in pigs. Based on these results we can make assumpations regarding the potential physiological functions of the NMB/NMBR system during reproduction.

Our studies show that NMB in pigs shares a common amino acid sequence NMB-10 (GNLWATGHFM) at the C-terminus with other species and that the mature peptide of NMB-32 (APLSWDLPEPRSRAGKIRVHPRGNLWATGHFM) [39] and the main protein structure of prepro-NMB shows high levels of homology with other animal species. Amino acid sequences from sequenced cDNA show that NMBR in pigs serves as a 390-amino acid protein with seven membrane spanning domains that are typical of G-protein coupled receptors [8]. Although NMB and NMBR mRNA distributions were studied across several vertebrates, results between different species were found to vary. NMB mRNA was observed most prominently in rat olfactory bulbs, dorsal root ganglion and dentate gyrus by *in situ* hybridization [3]. However, Northern blot analyses reveal NMB cDNA at high expression levels in the human hypothalamus, colon, and stomach and low levels in the human cerebellum, adrenals and pancreas [2]. The distribution of NMB mRNA found shows that it may act as a neuromodulator or neurotransmitter both centrally and peripherally. We found high levels of NMB mRNA in the olfactory bulb and hypothalamus, and these findings complement those of previous studies [2,3]. The expression of NMB mRNA indicated that it may act as neuromodulators or neurotransmitters both centrally and peripherally. In this exprement, we found that NMB mRNA was mainly expressed in CNS found among tissues, suggesting that NMB may have various functions in pigs. In our study, high levels of NMBR mRNA were mainly found in the peripheral tissues. However, NMBR mRNA was found to be weakly expressed in brain tissues, contradicting previous data on humans and mice [10,11]. Although our tissue expression results complement those of other studies, a few tissues showed discrepancies with other vertebrates. Thus, our results may guide further research on the multifunctional role of the NMB/NMBR system in the brain and on its other physiological functions.

Additional IHC analyses show that NMBR immunoreactive cells are broadly detected in several pig tissues and organs. NMBR immunoreactivity in the trachea, lungs, aorta and heart suggest that NMBR may be involved in the lung development/injury and blood pressure. In regards to the digestive system, NMBR staining was found in the parotid gland, liver, pancreas, esophagus, stomach, duodenum, jejunum and ileum, which are functionally associated with feeding, sucrose regulation, energy balance and gastrointestinal smooth muscles [20,24,25]. Meanwhile, our studies also showed that NMBR immunoreactivity levels were examined in urogenital organs, lymphatic organs and some glands. These results are consistent with NMBR gene mRNA expression patterns in these tissues. NMBR distribution patterns in the pituitary-adrenal gland and immune organs suggest that NMB may regulate stress response and immune response activity [40]. NMBR expression patterns found in the pituitary–thyroid suggest that NMB may play a key role in regulating pituitary-thyroid axis function and gene expression [27]. The presence of NMBR mRNA and protein in the pituitary, testis, ovaries and uterus suggests that NMB may play an important role in reproduction [16,29]. Further analyses must determine whether NMB can affect pig reproductive systems.

Thus, we also examined expression patterns of NMB and NMBR mRNA along the reproductive axis for female pigs across the estrous cycle and for male pigs at post-natal development stages. In regards to the hypothalamus, our results show that NMB and NMBR mRNA patterns were similar, with the highest levels of NMB and NMBR expression found during proestrus (though these levels were not significant compared to results the found during estrus). This suggests that NMB may control the release of GnRH through the regulation of NMBR secretion [29]. In the pituitary, we found relatively low levels of NMB and high levels of NMBR mRNA expression during estrus. This result supports the suggestion that NMB effects on the HPG axis stimulate the GnRH of the hypothalamus rather than directly acting on the pituitary [29]. Interestingly, peak NMB expression levels were observed during proestrus, whereas peak NMBR expression levels occurred during estrus. This suggests that NMB may regulate ovaries through its own receptor. Furthermore, we discovered various expression patterns of NMB and NMBR mRNA along the HPT axis of post-natal development stages. Hypothalamic mRNA expression levels of NMB and NMBR were found to be similar. Expression levels increased between postnatal day 30 and postnatal day 60 and decreased thereafter. Iwasa et al. found GnRH mRNA expression to change significantly with age in rats [41], and their results correspond roughly with NMB/NMBR change patterns. In the pituitary and testis, we found NMB and NMBR mRNA expression patterns differ entirely. Changes in NMB and NMBR expression levels may be responsible for pubertal activation and senescence in pig testis. All these data suggest that NMB plays an important role in the reproductive axis regulation. Further analyses of these molecular and morphological date may reveal additional physiological functions performed by the NMB/NMBR system.
